# Differentiation of renal masses with multi-parametric MRI: the de Silva St George classification scheme

**DOI:** 10.1186/s12894-022-01082-9

**Published:** 2022-09-03

**Authors:** Suresh de Silva, Kathleen R. Lockhart, Peter Aslan, Peter Nash, Anthony Hutton, David Malouf, Dominic Lee, Paul Cozzi, Fiona MacLean, James Thompson

**Affiliations:** 1 Faculty of Medicine, University of NSW, Kensington, NSW Australia; 2 Department of Radiology, I-MED Radiology Network, Ground Floor, 527-533 Kingsway, Miranda, 2228 Australia; 3 Department of Urology, St George Hospital, Kogarah, NSW Australia; 4 Department of Urology, Hurstville Private Hospital, Hurstville, NSW Australia; 5 Department of Anatomical Pathology, Sonic Healthcare, Ryde, NSW Australia

**Keywords:** Renal cell carcinoma, Renal tumours, Magnetic resonance imaging, Diagnostic accuracy, Classification

## Abstract

**Purpose:**

To develop a system for multi-parametric MRI to differentiate benign from malignant solid renal masses and assess its accuracy compared to the gold standard of histopathological diagnosis.

**Methods:**

This is a retrospective analysis of patients who underwent 3 Tesla mpMRI for further assessment of small renal tumours with specific scanning and reporting protocol incorporating T2 HASTE signal intensity, contrast enhancement ratios, apparent diffusion coefficient and presence of microscopic/macroscopic fat. All MRIs were reported prior to comparison with histopathologic diagnosis and a reporting scheme was developed. 2 × 2 contingency table analysis (sensitivity, specificity, positive predictive value (PPV) and negative predictive value (NPV)), Fisher Exact test were used to assess the association between suspicion of malignancy on mpMRI and histopathology, and descriptive statistics were performed.

**Results:**

67 patients were included over a 5-year period with a total of 75 renal masses. 70 masses were confirmed on histopathology (five had pathognomonic findings for angiomyolipomas; biopsy was therefore considered unethical, so these were included without histopathology). Three patients were excluded due to a non-diagnostic result, non-standardised imaging and one found to be an organising haematoma rather than a mass. Therefore 72 cases were included in analysis (in 64 patients, with seven patients having multiple tumours). Unless otherwise specified, all further statistics refer to individual tumours rather than patients. 52 (72.2%) were deemed ‘suspicious or malignant’ and 20 (27.8%) were deemed ‘benign’ on mpMRI. 51 cases (70.8%) had renal cell carcinoma confirmed. The sensitivity, NPV, specificity and PPV for MRI for detecting malignancy were 96.1%, 90%, 85.7% and 94.2% respectively, Fisher’s exact test demonstrated *p* < 0.0001 for the association between suspicion of malignancy on MRI and histopathology.

**Conclusion:**

The de Silva St George classification scheme performed well in differentiating benign from malignant solid renal masses, and may be useful in predicting the likelihood of malignancy to determine the need for biopsy/excision. Further validation is required before this reporting system can  be recommended for clinical use.

**Supplementary Information:**

The online version contains supplementary material available at 10.1186/s12894-022-01082-9.

## Introduction

A large proportion of renal masses are found incidentally since the adoption of widespread Computed Tomography (CT) and ultrasound (US), presenting a diagnostic and management dilemma. CT and US cannot reliably differentiate between benign and malignant renal tumours or malignant subtypes (impacting prognosis). As most identified masses are renal cell carcinomas (RCC), radical or partial nephrectomy is often considered gold-standard treatment. Surgery, however, involves significant morbidity and mortality risks, particularly in the elderly, the co-morbid (obesity, anticoagulation, previous abdominal surgery, inherited conditions predisposing to renal tumours, etc.) and those with impaired kidney function. Furthermore, up to 33% of excised or biopsied renal masses are benign or indolent, pathology proving in hindsight that the surgery was avoidable [1, 2].

Since malignancy may not be accurately predicted by conventional imaging, renal core biopsy is often used to confirm or exclude malignancy in patients where risks of surgery are near-prohibitive. However, unlike biopsy in other urologic tumours such as bladder and prostate, renal biopsy carries significant risks of life-threatening haemorrhage, injury to surrounding organs (liver, lung, spleen, bowel, blood vessels) and tumour seeding. A biopsy may be ‘non-diagnostic’ in 10–23% of cases and sampling error can underestimate the tumour grade within heterogenous RCCs [1, 2]. Not all patients are candidates for biopsy due to tumour location (e.g., a medial peri-hilar tumour), nature (cystic or small) or patient factors (single kidney, obesity, bleeding disorders or medications that increase bleeding risk). There is, therefore, a clinical need for a non-invasive method of improving diagnostic accuracy in renal tumour assessment.

Magnetic Resonance Imaging (MRI) has played an increasing role in assessment of urogenital system tumours including solid renal masses. This involves a multi-parametric approach assessing properties at both a macroscopic and microscopic level. Key properties include the T2 HASTE signal intensity (SI), the degree of enhancement post-contrast, evaluation of macroscopic and microscopic fat and calculation of the apparent diffusion coefficient (ADC). By microscopic fat we refer to the intra voxel coexistence of small amounts of fat and water, as opposed to bulk or macroscopic fat. The ADC value is calculated from protocols that include diffusion weighted imaging (DWI). DWI captures inherent differences in how tissues restrict water motion (Brownian motion). It is influenced by multiple factors including cellularity, cell membrane integrity, nuclear-to-cytoplasmic ratio and viscosity. The ADC value is effectively a measure of the ability of water molecules to move freely. As different renal tumour types vary in terms of their above structural properties, this represents a potential means of differentiation.

To our knowledge, there are very few published classification schemes which combine multiple MRI parameters to differentiate renal masses in a systematic format. We believe it is important to add to the body of literature in this regard.

The aim of our study was to develop an MRI classification scheme for solid renal masses and to assess its accuracy in differentiating benign from malignant tumours.

## Materials and methods

During the period June 2014–June 2019, all patients who had MRI imaging for solid renal masses at the same 3 Tesla (3 T) imaging facility and who subsequently had histopathological confirmation of diagnosis were evaluated retrospectively from a prospectively maintained database by the study radiologist (SDS). The scope of this study only included the those for whom MRI was clinically indicated. In total there were 75 renal masses. Five of these were diagnostic of lipid rich angiomyolipoma (AML) due to the presence of macroscopic fat on MRI without calcification. The study authors and treating urologists believe it would have been unethical to unnecessarily biopsy (given biopsy risks) due to the pathognomonic imaging findings. However, as the tumour types were diagnosed as benign with appropriate investigation and required no intervention, they were included in this study cohort. Three patients were excluded from analysis due to: non-diagnostic result on biopsy (not repeated; the patient opted for definitive focal ablation with cryotherapy), non-standardised imaging protocol and one was found to be an organising haematoma rather than a mass.

All patients underwent MRI on a 3 T Siemens Skyra (Siemens Healthineers, Erlangen, Germany) using a 30-channel body array placed over the pelvis and using the posterior coil elements of the in-table spine array (Table 1). Scans included the following conventional non-contrast breath-held sequences: axial in- and out-of-phase T1, axial and coronal 2D T2-weighted HASTE, and axial and coronal 3D fat-suppressed T1-weighted volumetric interpolated breath-hold examination (VIBE) sequence. All scans included dynamic contrast-enhanced VIBE sequences obtained following the administration of gadolinium. The contrast agent was administered as an IV bolus using a power injector (Bracco) followed by a 30-mL saline flush (both injected at 2 mL per second). Contrast dose was prescribed according to patient weight, and contrast-enhanced sequences were obtained at the corticomedullary (CM), nephrographic and excretory phases. All scans included a DWI sequence in the axial plane. DWI scans were a three-scan trace, monopolar with three diffusion directions. Diffusion b values were 50, 400 and 800, the DWI sequence used was 2D echoplanar, spin echo-free breathing with a scan time of ~ 4:30 min. Fat suppression was used, with a Repetition time (TR) of 6100 ms and Echo time (TE) of ~ 61 ms. ADC maps were obtained [3].

**Table 1:** 3-T MRI Renal Protocol

	Sequence						
Parameter	Axial T1-weighted In- and opposed-phase unenhanced 3D VIBE	Axial T2-weighted 2D HASTE	Axial T1-weighted fat-saturated unenhanced 3D VIBE	Axial T1-weighted fat-saturated contrast-Enhanced 3D VIBE	Coronal T1-weighted fat-saturated 3D VIBE	Coronal T2-weighted 2D HASTE	DWI^a^
Fat saturation	No	No	Yes	Yes	Yes	No	Yes, spectral attenuated inversion recovery
TR/TE	3.97/1.29	1600/95	4.15/2	4.15/2	3.44/1.29	1300/91	6000/59
Thickness (mm)	3	5	3	3	1.5	5	5
FOV (mm)	380	380	380	380	440	400	380
No. of slices	72	36	72	72	120	30	36
Matrix	320 × 240	320 × 203	320 × 195	320 × 195	384 × 270	256 × 256	192 × 116
Scan time	14 s	1 min 12 s	14 s	14 s	43 s	43 s	4 min 11 s
Delay (s)				40, 90, 300, 600 s	150, 540 s		

The contrast agent used for all sequences was 5 mL of gadobutrol (Gadovist, Bayer HealthCare)

*VIBE* volumetric interpolated breath-hold examination

^a^b values of 50, 400, and 800 s/mm^2^ were used

All scans included an axial 3D fat-suppressed Dixon T1-weighted volumetric interpolated breath-hold examination (VIBE Dixon) sequence, with a TR of 3.97, TE1 of 1.29 ms and TE2 of 2.52 ms. A 3D slab with 72 slices and a slice thickness of 3 mm, Field of view (FOV) 38 cm, was obtained. The Dixon method relies on acquiring an image when fat and water are in-phase and another when they are out- of-phase. When spins are out-of-phase, a black border is seen around organs surrounded by fat, such as the kidneys. This is chemical shift artefact. At 3 T, fat and water are in-phase at multiples of ~ 2.6 ms, and out-of-phase scans will be obtained at multiples of ~ 1.3 ms. The Dixon sequence can deliver four contrasts in one measurement [3].

**Fig. 1:**
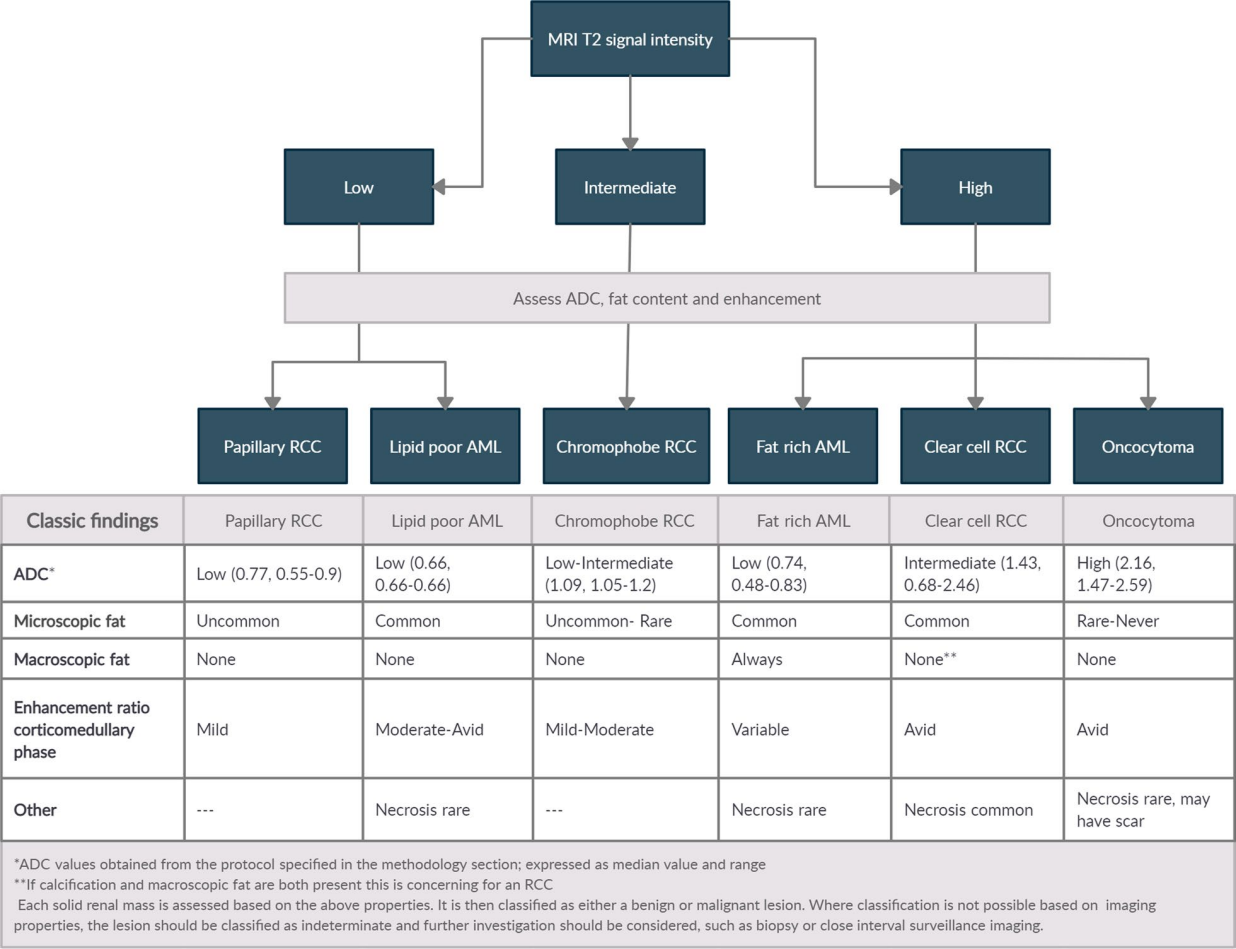
Renal MRI de Silva St George classification scheme

The imaging data was then reviewed by one sub-specialist abdominal radiologist with 17 years’ experience in evaluating body MRI imaging (SDS), applying the de Silva St George (dSG) classification scheme demonstrated in Fig. 1. This system reviews four key properties in determining the nature of solid renal masses. A fifth group of properties can also be used, consisting of the presence or absence of necrosis or a scar.

**Fig. 2:**
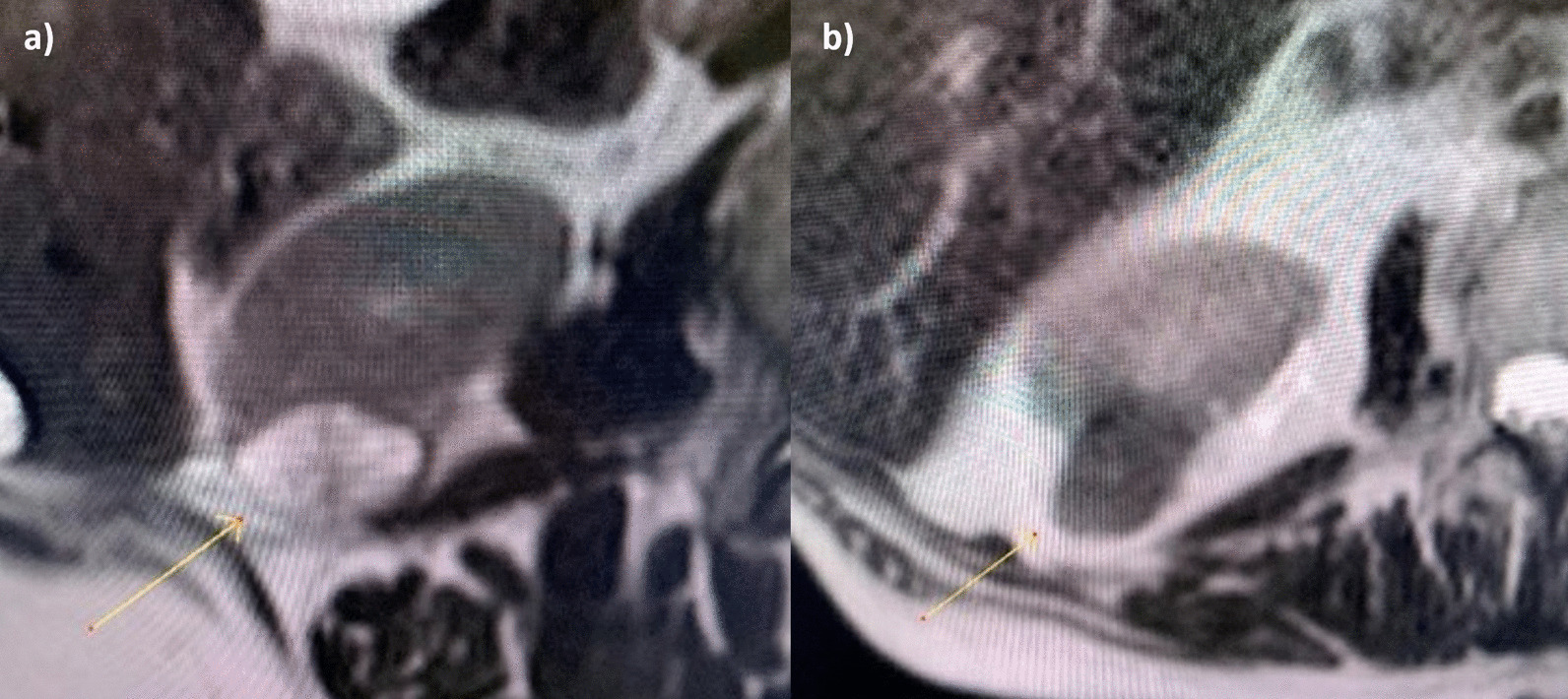
**a** Oncocytoma. Demonstrates increased signal intensity on T2 relative to the renal parenchyma. **b** Papillary RCC. Demonstrates decreased signal intensity on T2 relative to the renal parenchyma

The first key property is the predominant qualitative SI of the lesion on a non-fat suppressed T2 weighted sequence relative to the renal parenchyma. Based on this, lesions are divided into those that are hyper-intense, hypo-intense or iso-intense. (Fig. 2).

**Fig. 3:**
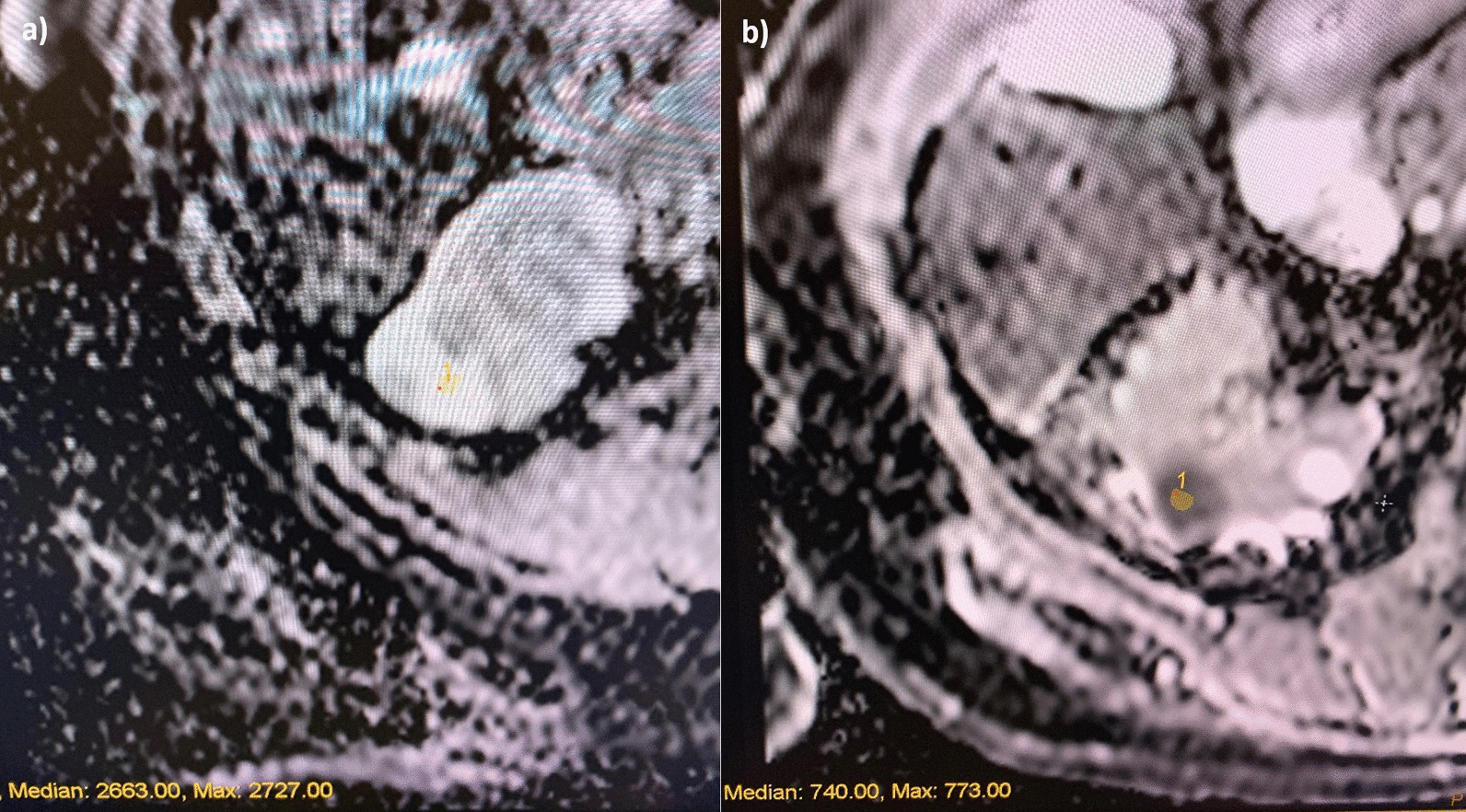
**a** High ADC number of 2.66 in a patient with an Oncocytoma. **b** Low ADC number of 0.74 in a patient with a Right papillary cell carcinoma

The second key property is the quantitative ADC measurement. The ADC map is reviewed for the qualitative ADC most representative of the renal mass. A 2-D region of interest (ROI) of up to 50mm^2^ was then measured in conjunction with the T2 weighted imaging and post contrast images to ensure placement of the ROI over solid tumour and not cystic/necrotic portions. In masses where qualitatively there were two ADC values equally represented in the mass, two ROI were measured, one in each region, and their mean calculated to provide tumour ADC value (Fig. 3). With regard to enhancement ratios, the region of the tumour which was most reflective of the overall enhancement of the tumour was measured.

**Fig. 4:**
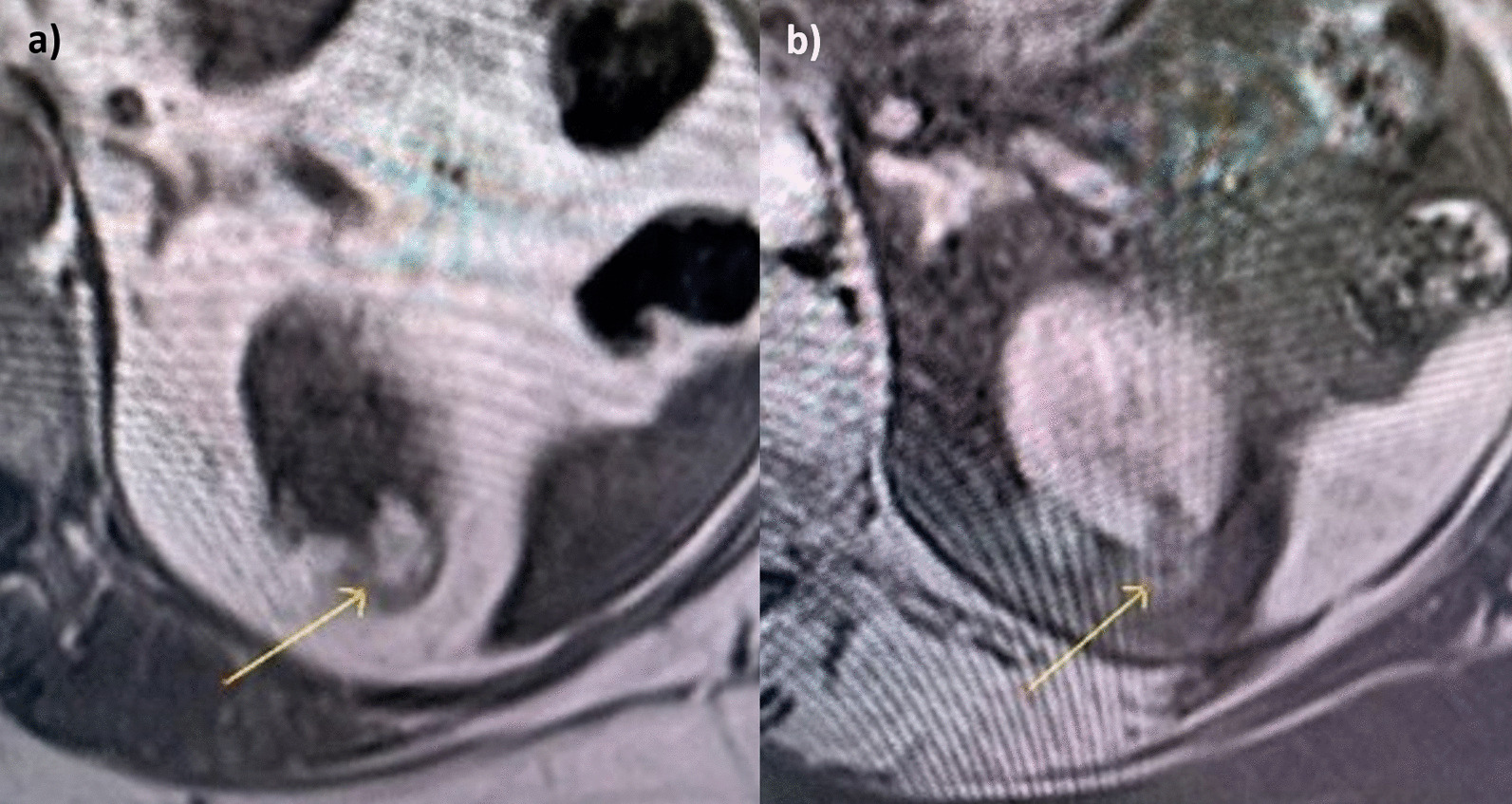
**a** T1 Weighted sequence non-fat suppressed demonstrates bright signal intensity in an angiomyolipoma due to the presence of fat. **b** T1 weighted sequence fat suppressed demonstrates low signal intensity in the same mass due to the suppression of the signal intensity of the macroscopic fat

**Fig. 5:**
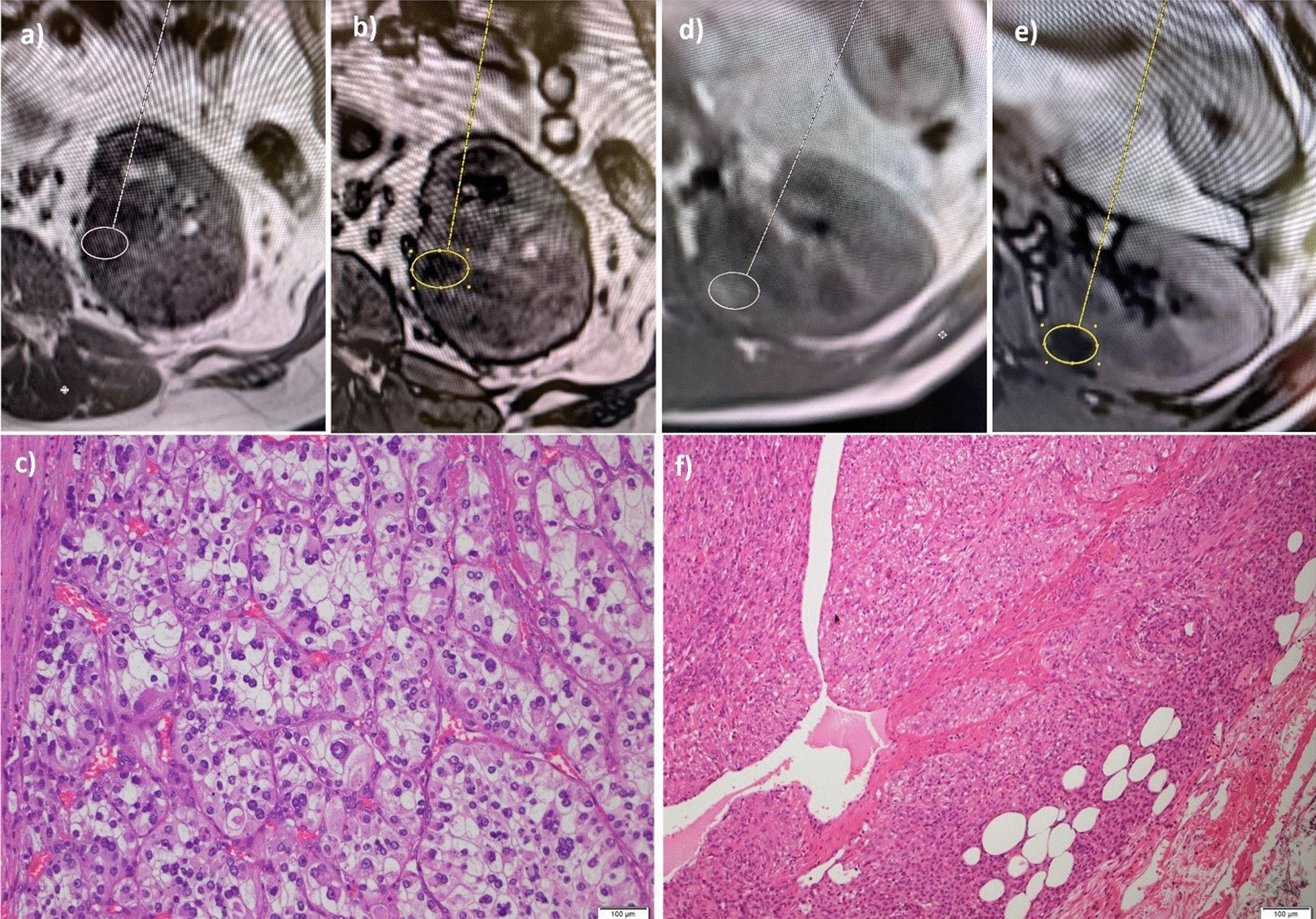
**a** RCC; In phase T1 weighted sequence demonstrates some bright signal in the lesion. **b** RCC; Opposed phase T1 weighted sequence demonstrates loss of signal in the lesion consistent with microscopic fat. Chemical shift index calculated at 25%. **c** Clear cell RCC. **d** AML; T1 in phase image demonstrates bright signal in the lesion, **e** AML; T1 opposed phase image demonstrates significant drop of signal consistent with microscopic fat. The chemical shift index was calculated at 67%, **f** Adipocytes demonstrated in an AML

The third key property involves assessing the presence or absence of macrosocopic and microscopic fat. Macroscopic fat can be assessed by loss of signal in the mass on fat suppressed sequences (Fig. 4). For microscopic fat, both the in-phase and opposed-phase T1-weighted sequences were assessed for any drop of signal; microscopic fat is deemed present by a comparative drop of signal in the opposed phase images. In assessing microscopic fat, the area with the greatest qualitative drop between the in and opposed phase gradient echo sequences was measured in the ROI. In chemical shift imaging, decrease in SI on opposed phase imaging is a function of the ratio of lipid content to the total amount of tissue in each voxel [4]. Lesions with no drop of signal were interpreted as not containing microscopic fat (Fig. 5). In lesions with microscopic fat, the region with the greatest drop of signal was analysed to assess the amount of microscopic fat with the following equation to calculate the chemical shift index (CSI):

$${\text{CSI}} = \, \left( {{\text{SI in phase}} - {\text{ SI opposed phase}}} \right){\text{/ SI in phase}}\; \, \times \; \, 100$$

**Fig. 6:**
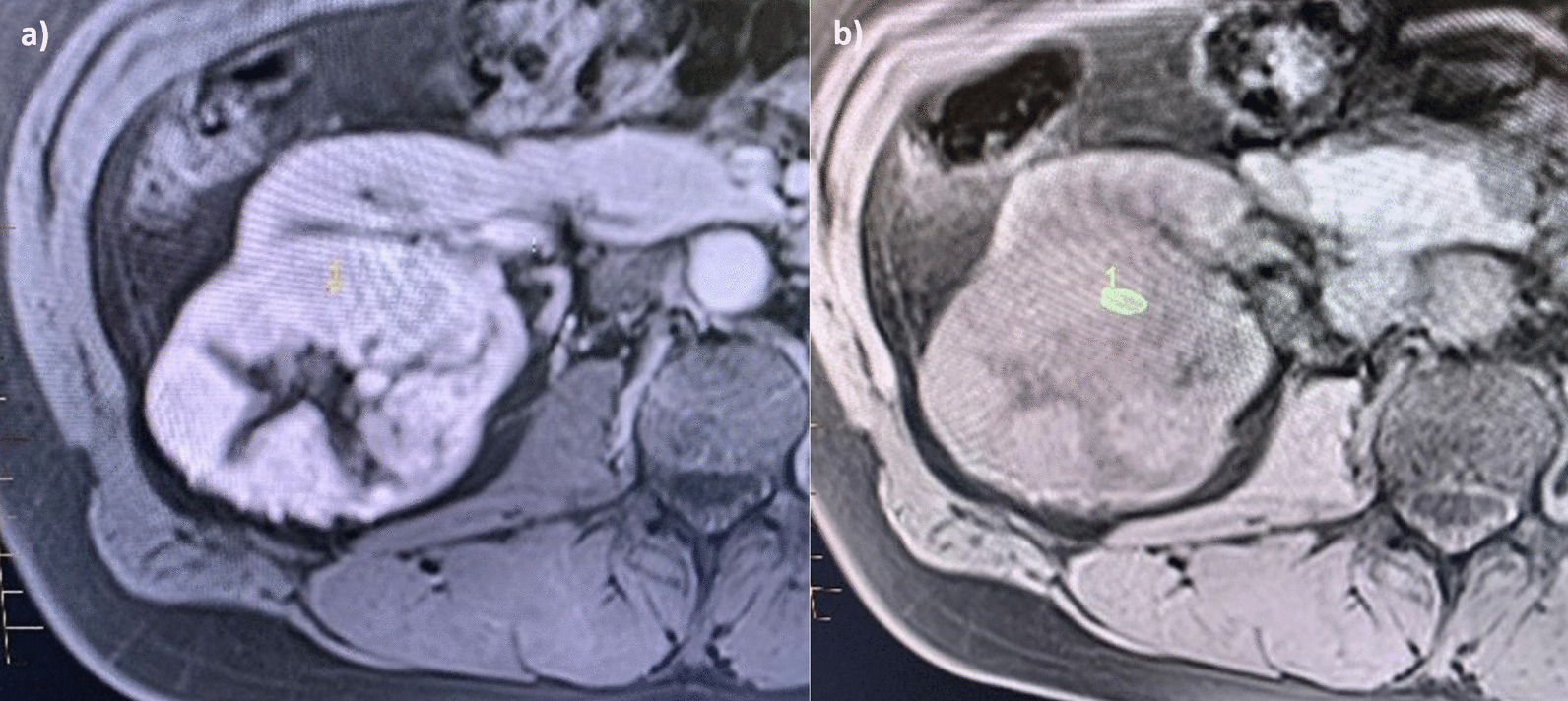
**a** Clear cell RCC. **b** There is avid enhancement post contrast with the enhancement ratio calculated at 204 (percentage SI change)

The fourth key property involves assessing the enhancement ratio of the lesion in the CM phase post-contrast administration (Fig. 6). A ROI (most representative of the overall enhancement of the solid mass) of between 50 and 100 mm^2^ was placed in the lesion pre contrast and in the same position post-contrast in the CM phase. Qualitative and quantitative assessment of the degree of enhancement was made based on the following equation:

$${\text{Contrast enhancement ratio CM phase }} = \, \left( {{\text{SI CM phase }} - {\text{ SI Pre Contrast phase/ SI Pre Contrast phase}}} \right)\; \, \times \; \, 100$$

All histopathology specimens were received intact in formalin. After appropriate fixation and sampling, sections were prepared using standard techniques and stained with haematoxylin and eosin. In cases which required immunoperoxidase stains, these were prepared using validated criteria on a Ventana platform. The slides were assessed by subspecialty histopathologists in a single high volume genitourinary pathology practice, blinded to the radiological diagnosis. In cases in which there was diagnostic uncertainty, the material was re-reviewed by a highly experienced histopathologist with sub-specialised in renal uro-pathology (FM).

The study received approval by the institutional research ethics and governance system (REGIS).

Initial statistical analysis comprised 2 × 2 contingency table analysis, enabling the calculation of sensitivity, specificity, negative and positive predicted values. Fisher Exact Test was used to determine the association between suspicion of malignancy on MRI and confirmed malignancy on histopathology. The pre- and post-test probability of malignancy were then calculated for a ‘positive’ (equivocal/malignant) and ‘negative’ (benign) MRI result. Where MRIs of tumours demonstrated equivocal findings or two possible diagnoses where one may be benign and one malignant, a diagnostic decision was made for the purposes of analysis based on available characteristics and the lesion was then dichotomously categorised for 2 × 2 contingency table analysis.

Further analysis was based on descriptive statistics with exact matching. As some of the radiological diagnoses involved two predictions to compare with the gold standard histopathology (11 lesions), this analysis was subdivided into two concepts (one exact match prediction vs two exact matching prediction) to distinguish for these effects more profoundly. 95% confidence interval was calculated and *p* value < 0.05 was considered statistically significant. The cases with two diagnoses were felt to be important in the mix of cases provided for this histologic analysis and were included, as this represents a likely clinical scenario.

## Results

72 tumours were included in analysis. Clinical, radiologic and pathologic characteristics of the population are summarised in Table 2. 52/72 (72.2%) were deemed ‘suspicious or malignant’ and 20/72 (27.8%) were deemed ‘benign’ on mpMRI. 51/72 (70.8%) cases had renal cell carcinoma confirmed on histopathology. 7 patients had multiple tumours. Of those masses which had histopathological gold standard diagnosis, 39.6% were diagnosed with partial nephrectomy, 45.3% with radical nephrectomy and 15.1% with core biopsy. One patient had a non-diagnostic biopsy (excluded as described in Methods).

**Table 2:** Clinical, MRI and pathologic characteristics of the 72 cases analysed (unless otherwise specified)

Characteristic	Value
Mean (median) age (years)	66.52 (68)
Mean (median) tumour diameter (mm)	40.56 (32)
Number of tumours per patient (% of total 75 cases)	
1	57 (76%)
2	4 (10.67%)
> 2	3 (13.33%)
Sex Male:Female ratio of total 75 cases (% Male)	38:37 (50.67%)
Equivocal or suspicious for malignancy on MRI (%)	11.1%
Signal on T2 (mean/median(range))	1.03/0.96 (0.67–1.54)
Micro Fat detected (%)	37.5
Median enhancement ratio- Corticomedullary phase (%)	
Clear cell RCC	206
Papillary RCC	32
Chromophobe RCC	110
Malignancy on final histopathology (% total)	70.9%
Clear cell RCC	35 (48.6%)
Papillary RCC	11 (15.3%)
Chromophobe RCC	5 (6.9%)
Benign on final histopathology (%)	29.1%
AML	10 (13.9%)
Oncocytoma	11 (15.3%)

### Contingency (2 × 2) table analysis

The sensitivity, negative predictive value, specificity and positive predictive value for MRI for detecting malignancy were 96.1% (95% CI 86.5–99.52%), 90.0% (95% CI 69.6–97.3%), 85.7% (95% CI 63.7–97.0%) and 94.2% (95% CI 85.1–97.9%) respectively (see Table 3). Given a pre-test probability of 70.8% in this population (prevalence), a positive (or equivocal/suspicious) MRI increased the risk of malignancy to 92.7% (PPV), whilst a negative (benign) MRI decreased the risk to 10.0% (100% minus NPV). This reflects an overall calculated accuracy of 93.1% (95% CI 84.5–97.7%). The Fisher Exact Test demonstrated a strong association between suspicion of malignancy on mpMRI and proven malignancy on histopathology, *p*-value < 0.00001.

**Table 3:** Binary chi square relationship between MRI and histopathology

	Histopathology positive (malignant)	Histopathology negative (benign)	Total
MRI findings positive (suspected malignancy)	49	3	52
MRI findings negative (benign appearance)	2	18	20
Total	51	21	72

The frequency/volume relationship of MRI category (benign, equivocal, malignant) with tumour sub-type (clear cell RCC, papillary RCC, oncocytoma, AML, other) is described in Additional file 1: Table S1. The individual clinical, MRI and pathologic characteristics for false positives and negatives on MRI are described in Additional file 1: Table S2.

### Single matching analysis

57 of 61 lesions were correct with single match prediction compared to histopathological diagnosis; four were incorrect (93% vs 7%). Of the 61 tumours, 42 were classified as malignant and 19 as benign. Of the four incorrectly matched lesions, three were incorrectly matched as aggressive. The final, although correctly categorised as benign, had incorrect tumour type diagnosis. The incorrect predicted diagnosis of three aggressive tumours was deemed clinically significant so we included this as a manual adjustment, and the benign case was listed in the correct predicted diagnosis, resulting in 58/61 deemed correct (95% vs 5%).

### Two matching analysis

We repeated the same process for the 11 lesions which had two possible radiological diagnosis predictions. Out of the 11 lesions there were 9 correct matches based on at least one radiological predicted diagnosis. Two cases had neither diagnosis correct. Of these two incorrect lesions, one was correctly matched as malignant whilst one was incorrectly matched as benign. The missed malignant diagnosis was deemed clinically significant and the analysis was adjusted, resulting in correct diagnostic prediction in 10 of the 11 cases (16% vs 2%).

### Overall summary of lesion-matching analysis

Taking the overall summary of both the adjustments made for single and two matching predictions, 68/72 (94%) correctly matched lesions compared to 4/72 (6%) incorrectly matched in determining whether the lesion was benign or malignant (95% CI 76.6–93.0%, *p* < 0.0001).

## Discussion

Our results demonstrate that multi parametric MRI with proposed dSG classification scheme may differentiate benign from malignant solid renal masses with a high degree of sensitivity and specificity. This may be useful in determining the need for biopsy/excision in equivocal cases, especially those with relative contraindications.

The dSG classification scheme begins with the assessment of the T2 SI of the lesion. Based on this, lesions are divided into those that demonstrate generally high SI compared to the normal renal cortex and those demonstrating lower SI. Clear cell RCCs typically show high T2 SI, as do oncocytomas [5–7]. AMLs will vary in T2 SI depending on the fat content in the lesion, however typical AMLs containing macroscopic fat appear relatively hyperintense [6]. The diffusion characteristics and ADC value are then used to differentiate these tumour types further.

Taouli et al. found that oncocytomas had significantly higher ADC values than those of other solid RCCs and AMLs [8, 9]. A meta-analysis of studies performed by Lassel et al. found that the ADC number could be used to differentiate oncocytomas from potentially malignant tumours [9]. Our working group has also confirmed this finding on assessment of tumours with a high T2 SI and using the imaging protocol described in the methodology section found that the median ADC value for oncocytomas was 2.16 [3]. Clear cell RCCs have an intermediate ADC value confirmed by several groups including that of Wang et al., who found that on a 3 T MRI (using b values of 0 and 800), the mean ADC value was 1.698 [10]. Our working group using the protocol specified in Methods found the median ADC value to be 1.50 [3]. AMLs have the lowest ADC value of renal tumours (0.69), significantly lower than those of non-papillary RCCs (*p* < 0.048) [5].

Following assessment of ADC values, the next characteristic assessed is the presence or absence of macroscopic and microscopic fat. The unequivocal presence of macroscopic fat in a lesion is considered confirmatory of a typical fat rich AML [11, 12]. Although there have been reported cases of RCC with significant fat density, with few exceptions they have also had calcification- so if both macroscopic fat and calcification are present the lesion should be presumed to be malignant and treated accordingly [11, 13]. Several studies have shown the presence of microscopic fat in a lesion most commonly occurs in AMLs and clear cell RCCs (60%) and is rarely seen in oncocytomas [4, 14, 15]. Contrast enhancement is then assessed in the corticomedullary phase.

In terms of contrast enhancement in the CM phase, typical AMLs containing macroscopic fat can show different degrees of enhancement depending on the amount of vascularised tissue content they contain [5]. Clear cell RCCs and oncocytomas are generally avidly enhancing [16]. Sun et al. found that the contrast enhancement ratio in the CM phase for clear cell RCCs had a mean of 205.6 [17].

Although these properties are often adequate in differentiating tumour types, (particularly clear cell RCCs, fat rich AMLs and oncocytomas) other features may be useful. The appearance of a central scar may support the diagnosis of an oncocytoma, but can also be seen in RCCs from necrosis or cystic change [6]. Central necrosis is common in clear cell RCCs but is rare in benign tumours [5]. Oncocytomas may show delayed enhancement after the administration of gadolinium based contrast material [5].

Although AMLs, oncocytomas and clear cell RCCs usually show increased T2 SI, in our authors’ experience they may occasionally show intermediate SI; therefore in this instance intermediate SI should not preclude the diagnosis and other properties should be considered carefully.

The next category of tumours to consider are those demonstrating low SI relative to the renal cortex. Papillary RCCs usually show low SI relative to renal cortex on T2, as is the case with lipid poor AMLs, which do not contain any macroscopic fat and will appear low SI due to the abundance of smooth muscle [5, 6, 18, 19]. They can often be differentiated as lipid poor AMLs commonly contain microscopic fat, significantly more often than in papillary RCCs [4, 14, 15]. Our working group also established that the mean CSI for lipid poor AMLs was 73%, significantly higher than any other renal tumour type [14].

The enhancement ratio can also be very useful in the differentiation of these two tumour types. Papillary RCCs only demonstrate low levels of enhancement, with Sun et al. evaluating the mean contrast enhancement ratio in the CM phase at only 32% [16, 17, 20, 21]. Lipid poor AMLs, on the other hand, enhance moderately to avidly in the CM phase [6, 17, 22]. DWI/ADC assessment is not useful in differentiating the two tumour types as although the ADC of papillary RCCs is the lowest of all RCCs, it can be similar to that of AMLs [8, 10]. Our working group found that the mean ADC value for AMLs was 0.69 while that for papillary RCCs was 0.76 [3].

The third category of tumours based on the T2 SI are those that are intermediate or similar to the renal cortex. This consists essentially of chromophobe RCCs, which although variable, are commonly intermediate to low SI [6]. Enhancement in the CM phase is intermediate, being less than that of clear cell RCCs but greater than that of papillary RCCs [6, 23]. Sun et al. [17] found that the contrast enhancement ratio in the CM phase for chromophore RCCs was a mean of 109.9. They have been reported to have ADC values that are lower than clear cell RCCs, and generally higher than papillary RCCs [6, 10]. The ADC value is low to intermediate with our working group evaluating the mean ADC value at 1.11 [3]. They do not commonly contain microscopic fat. Cystic change and necrosis are uncommon features, even when the lesions are large [6].

As indicated by our results, we believe that this classification scheme can lead to the differentiation and classification of solid renal masses as either oncocytomas, fat rich or lipid poor AMLs (benign) or clear cell, papillary or chromophobe RCCs (malignant). In those cases where imaging findings are not conclusive, we would propose these be classified as indeterminate- requiring biopsy, excision or close interval surveillance imaging. In our study (see Results), 11 cases had two diagnoses postulated, which based on this system would have been classified as indeterminate.

Our study has several limitations. Firstly, the study was retrospective in design, although the prospective MRI reports were used and all consecutive eligible patients were included, reducing both selection and reporting bias. Images were reviewed by only one sub specialist abdominal radiologist. Further studies are required to assess for inter-reader variability. Secondly, although the qualitative assessment of the ADC/DWI is useful, the quantitative assessment is a key component of lesion characterisation. This can be vendor specific; a larger study including other vendors will be required for standardisation of the ADC values. If the dSG classification scheme is utilised for classifying solid renal masses this may lead to a greater degree of caution in reporting (and increased proportion of indeterminate lesions) if management is determined based on this system, as observed when prostate MRI was utilised to determine need for biopsy. In future prospective studies to mitigate the risk of introducing bias only one diagnosis would be proposed for each case. This however has been addressed in the management system proposed as these lesions will be considered indeterminate prompting further investigation/management. Additionally, it is notable that only a small number of papillary and chromophobe RCCs were included in the cohort. This likely reflects their low prevalence, however necessitates future studies with larger populations to validate these findings.

In conclusion, we believe that the dSG classification scheme is a relatively simple system which draws upon common readily available imaging properties for multi parametric MRI in the medical literature, which is efficacious in the differentiation of benign from malignant solid renal lesions. It also assists in confirming a management strategy for solid renal masses as those lesions that cannot be confidently identified as benign or malignant should be considered as indeterminate with a view towards biopsy or close interval surveillance imaging. Although larger studies are required to validate these findings, this classification scheme could have significant utility in the management of solid renal masses.

## Supplementary Information

**Additional file 1.** **Table S1**: Categorical relationship between MRI and histopathological sub-type. **Table S2**: Clinical, MRI and histopathological characteristics of cases with false positive/ negative MRIs.

## Data Availability

The data analyses generated are available within the presented study but specific datasets are kept confidential due to their nature of including clinical and radiological details. Further information regarding dataset analysis may be available from the corresponding author upon request.

## Abbreviations

MRI: Magnetic resonance imaging
DWI: Diffusion weighted imaging
ADC: Apparent diffusion coefficient
RCC: Renal cell carcinoma
CT: Computed tomography
T: Tesla
ROI: Regions of interest
ANOVA: Analysis of variance
AML: Angiomyolipoma
US: Ultrasound
MpMRI: Multiparametric magnetic resonance imaging
VIBE: Volume interpolated breathhold examination
2D: Two-dimensional
TR: Repetition time
TE: Echo time
FOV: Field-of-view
EPI: Echo planar
Hz/Px: Hertz per pixel
CKD: Chronic kidney disease
