# Supplementary material for: Differentiation of renal masses with multi-parametric MRI: the de Silva St George classification scheme
Source: BMC Urol. 2022 Sep 3;22:141. doi: 10.1186/s12894-022-01082-9 (PMC9441035; doi:10.1186/s12894-022-01082-9)
Supplement: Supplementary file 1 — Additional file 1. Table S1: Categorical relationship between MRI and histopathological sub-type. Table S2: Clinical, MRI and histopathological characteristics of cases with false positive/ negative MRIs. [file 12894_2022_1082_MOESM1_ESM.pdf]

Supplementary Table 1: Categorical relationship between MRI and histopathological sub-type

	Histopathology					
MRI		**AML**	**Oncocytoma**	**Clear cell RCC**	**Papillary RCC**	**Chromophobe RCC**
	**Benign**	8	10	2	0	0
	**Equivocal**	2	1	3	2	0
	**Malignant**	0	0	30	9	5
	**Total**	10	11	35	11	5

Supplementary Table 2: Clinical, MRI and histopathological characteristics of cases with false positive/ negative MRIs

**Variable**	**Case**									
	1	2	3	4	5	6	7	8	-9	10
**Age (years)**	57	29	39	79	65	82	73	69	62	79
**Size (mm)**	20	31	24	22	37	30	37	18	14	35
**Number of tumours**	1	1	1	1	1	1	1	1	1	1
**T2**	1.7	1.8	1.07	0.67	1.23	1.35	1.54	0.76	0.79	0.84
**Micro fat**	absent	absent	absent	absent	present	absent	absent	present	absent	present
**ADC**	2.24	2.46	0.66	Not interpretable	2.1	1.68	2.25	1.47	Not interpretable	0.89
**Overall impression MRI**	benign	benign	equivocal/ suspicious	equivocal/ suspicious	equivocal/ suspicious	equivocal/ suspicious	equivocal/ suspicious	equivocal/ suspicious	equivocal/ suspicious	equivocal/ suspicious
**Histo type**	Clear cell RCC	Clear cell RCC	AML	AML	Clear cell RCC	Clear cell RCC	Clear cell RCC	Oncocytoma	Papillary RCC	Papillary RCC
